# Long Noncoding RNAs: Emerging Players in Medulloblastoma

**DOI:** 10.3389/fped.2019.00067

**Published:** 2019-03-14

**Authors:** Pietro Laneve, Jessica Rea, Elisa Caffarelli

**Affiliations:** ^1^Institute of Molecular Biology and Pathology, National Research Council, Rome, Italy; ^2^Department of Biology and Biotechnology, Sapienza University of Rome, Rome, Italy

**Keywords:** nervous system, pediatric tumor, medulloblastoma, long noncoding RNAs, oncogenes, tumor suppressors, diagnostic biomarkers, therapeutic targets

## Abstract

Central Nervous System tumors are the leading cause of cancer-related death in children, and medulloblastoma has the highest incidence rate. The current therapies achieve a 5-year survival rate of 50–80%, but often inflict severe secondary effects demanding the urgent development of novel, effective, and less toxic therapeutic strategies. Historically identified on a histopathological basis, medulloblastoma was later classified into four major subgroups—namely WNT, SHH, Group 3, and Group 4—each characterized by distinct transcriptional profiles, copy-number aberrations, somatic mutations, and clinical outcomes. Additional complexity was recently provided by integrating gene- and non-gene-based data, which indicates that each subclass can be further subdivided into specific subtypes. These deeper classifications, while getting over the typical tumor heterogeneity, indicate that different forms of medulloblastoma hold different molecular drivers that can be successfully exploited for a greater diagnostic accuracy and for the development of novel, targeted treatments. Long noncoding RNAs are transcripts that lack coding potential and play relevant roles as regulators of gene expression in mammalian differentiation and developmental processes. Their cell type- and tissue-specificity, higher than mRNAs, make them more informative about cell- type identity than protein-coding genes. Remarkably, about 40% of long noncoding RNAs are expressed in the brain and their aberrant expression has been linked to neuro-oncological disorders. However, while their involvement in gliomas and neuroblastomas has been extensively studied, their role in medulloblastoma is still poorly explored. Here, we present an overview of current knowledge regarding the function played by long noncoding RNAs in medulloblastoma biology.

## Introduction

Medulloblastoma (MB), with an estimated 5000–8000 cases/year worldwide (1, 2), is an aggressive tumor arising in the cerebellum. It mainly affects children and is a major cause of mortality in pediatric oncology (3). While the previous classification of MB by the World Health Organization (WHO) was largely based on histological features (4), the new classification in 2016 exploited molecular parameters to catalog the large variety of tumors of the Central Nervous System (CNS) (5). Rational molecular-based classification was supported by the advancement of sequencing technologies allowing extensive genomic/transcriptomic studies. This classification benefits from the integration between histological and molecular parameters and led to no longer considering MB as a unique pathology. Several subclasses of MB have been unveiled, each displaying dysregulated genes—the driver genes—altered by single nucleotide mutations, somatic copy-number aberrations, or by defects in transcriptional or post-transcriptional gene regulation.

In this review, we highlight the link between MB tumors and the emerging class of regulatory long noncoding RNAs (lncRNAs), and their potential as promising cancer biomarkers and novel therapeutic agents.

### Medulloblastoma

Recent genomic and transcriptomic analyses on a large cohort of primary tumors assigned MBs to four molecularly distinct subgroups (6, 7). They include the extensively characterized WNT and SHH subgroups, and the Group 3 (G3) and Group 4 (G4), whose pathogenesis and signaling pathways are poorly defined.

### WNT

Approximately 10% of all MB patients belong to this subgroup, characterized by the most favorable prognosis with 95% of survival (7, 8). WNT tumors, which exhibit classic histology, are recognizable by a WNT gene expression signature. Nuclear accumulation of β-catenin is considered a biomarker for WNT signaling pathway activation. This subgroup often carries heterozygous *TP53* mutations, as well as mutations in the DEAD-box helicase gene *DDX3X* and in chromatin modifiers genes, such as *SMARCA4* and *CREBBP*, indicating the implication of altered epigenome in the development of this disease. Integration of gene expression and DNA methylation profiles indicated that WNT subgroup comprises at least two subtypes, WNTα, mainly enriched for children and characterized by monosomy 6, and WNTβ, including mainly adults without monosomy 6 (9).

### SHH

SHH MBs represent approximately 30% of all MB cases, characterized by an intermediate prognosis, with survival rates ranging from 60 to 80% (7). SHH tumors, mainly exhibiting desmoplastic histology, display an aberrant activation of the SHH signaling, due to mutations of negative regulators of SHH pathway, such as *PTCH1* and *SUFU*, and copy number aberrations of SHH target genes, such as *MYCN* and *GLI2* (7). *TP53* mutations are found in about 30% of childhood SHH MBs and are associated with extremely poor outcomes. Recent analyses suggest that SHH subgroup consists of four distinct subtypes. It includes SHHα, enriched for *MYCN* and *GLI2* amplifications, with the worst prognosis; SHHβ, harboring *PTEN* gene deletions and frequently metastatic; SHHγ displaying scarce copy number aberrations and SHHδ, that is enriched for *TERT* gene promoter mutations and has a favorable prognosis (9).

### Group 3

G3 is the most aggressive subgroup accounting for about 25% of all MBs, about half of them being metastatic at diagnosis (10). These tumors display a MYC signature, being characterized by amplification of the *MYC* proto-oncogene and exhibiting aberrant *MYC* expression in almost all cases (7). G3 shows intra-tumoral heterogeneity, including three further subtypes: G3α and G3β with a more favorable prognosis compared to G3γ, which frequently harbors increased *MYC* copy number (9).

### Group 4

G4, the most common subtype, accounts for 35% of all MBs. These tumors are often metastatic at diagnosis and have intermediate prognosis. It is the most enigmatic subgroup, characterized by a neuronal gene expression signature, resembling that of glutamatergic neurons (7). Common alterations pertain to inactivating mutations in *KDM6A* gene, duplication of *SNCAIP* gene, and amplification of *MYCN* and *CDK6* proto-oncogenes. G4 has been re-classified into G4α, characterized by *MYCN* and *CDK6* amplifications, G4β, strongly enriched for *SNCAIP* duplications and putative *PRDM6* overexpression, and G4γ enriched for focal *CDK6* amplification (9).

## Long Noncoding RNAs

RNA is considered as the most “rediscovered” biological macromolecule (11, 12) since, starting from the informational role assigned to mRNAs in 1961 (13, 14), novel unexpected functions have been attributed to RNA in the last three decades. In the 1980s, its capacity to catalyze biochemical reactions was associated with its ability to fold into complex tridimensional structures (15). In the early 1990s, regulatory functions were attributed to two long RNAs lacking protein-coding capacity, *H1*9 (16, 17), and *XIST* (18, 19). Since then, a huge number of noncoding RNAs, both short and long in size, was discovered in parallel with the finding that more than half of the transcriptome encodes non-proteinogenic transcripts. Among them, the lncRNAs number in the tens of thousands and include also circular RNAs, covalently closed RNA circles derived from back-splicing of linear transcripts (20). LncRNAs are >200 nucleotides and represent very versatile molecules for their unique ability to specifically recognize both nucleic acids and protein partners via base-pairing and modular tridimensional structures, respectively. They are flexibly involved in important biological processes, such as development, cell differentiation and growth, thanks to their main functions of gene expression regulators and the genome structure architects.

### Mechanisms of Action

LncRNAs may be engaged in fine-scale modulation of gene expression as well as in large-scale control of developmental programs. They may act through a variety of mechanisms, depending on their cellular localization. Some of them are exclusively localized in the nucleus, others in the cytoplasm, others change their localization during development or differentiation, and still others show both localizations. In the latter case, a single lncRNA might have multiple molecular functions.

#### Nuclear lncRNAs

**Figure 1:**
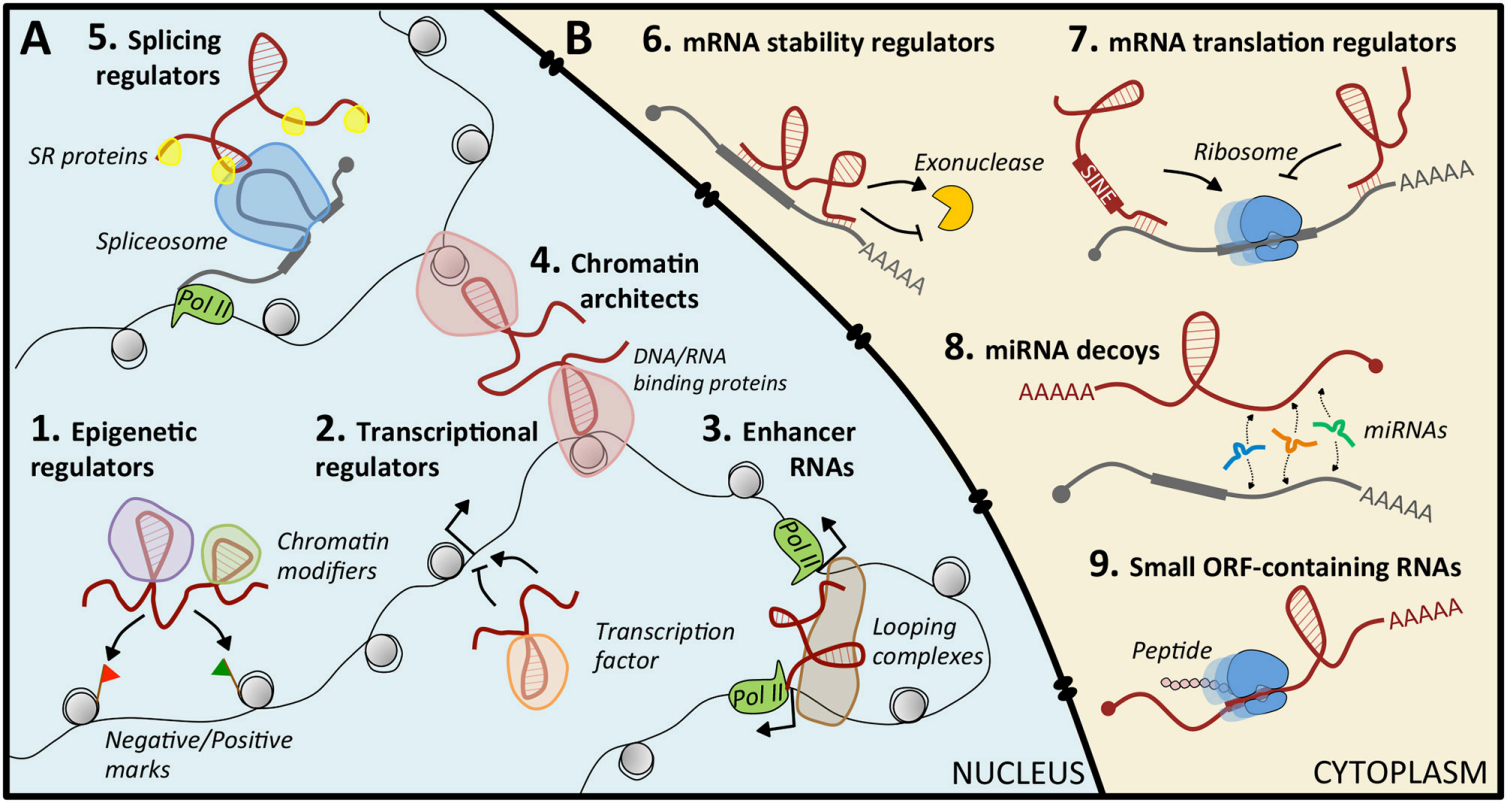
Mechanisms of action of lncRNAs. **(A)** In the nucleus, lncRNAs can regulate gene expression by guiding epigenetic (1) or transcription (2) factors to specific loci, by acting as enhancers (3), by structuring the three-dimensional conformation of chromatin (4), or by recruiting splicing factors (5). **(B)** In the cytoplasm, they act by modulating mRNA stability (6) or translation (7), by sequestering miRNAs (8), or by coding small peptides (9).

Nuclear lncRNAs (Figure 1A) can be found in the nucleoplasm or associated with chromatin (21). Typically, these latter are supposed to control protein-coding gene expression at the epigenetic level by recruiting chromatin modifiers to specific genomic loci. This is achieved through their scaffolding activity, by which they interact simultaneously with distinct protein complexes, and through their capability to act as “molecular guides,” that ensure the specificity of target recognition (21). This function can be carried out in *cis* or in *trans*. The *cis*-acting RNAs are typically low-abundant, and regulated genes are located in the proximity of their transcription site; *trans*-acting RNAs are more abundant and can modulate the expression of genes at independent loci (21). Notably, perturbations of the epigenetic regulation were recognized as causative of malignancies (22), and some cancer-related lncRNAs, such as *XIST* (23), *HOTAIR* (24–26), *NBAT* (27), and *LINC-PINT* (28), were reported to direct epigenetic modifications (29).

Nuclear lncRNAs can also act as regulators of transcriptional programs, by recruiting transcription activators or repressors to specific loci (30, 31), as enhancer RNAs that exert enhancer-like functions (32, 33), as chromosome architects and nuclear organizers that contribute to the formation of specific sub-nuclear structures (21, 34, 35), or as regulators of alternative splicing (36).

#### Cytoplasmic lncRNAs

Cytoplasmic lncRNAs (Figure 1B) regulate gene expression at the post-transcriptional level, often exploiting their sequence complementarity with transcripts deriving from the same genomic locus or from independent loci. Upon specific target recognition, they are able to modulate mRNA stability, both positively as *BACE1-AS* (37) and *TINCR* (38), and negatively as ½*-sbsRNAs* (39), or translation, as *lincRNA p21* (40). Another role is that of decoys for microRNAs (miRNAs): in this case, the lncRNA functions as a competing endogenous RNA (ceRNA) that sequesters miRNAs from their mRNA targets, causing translational de-repression. This activity is based on regulatory crosstalk between multiple transcripts (41, 42). Notably, lncRNA-mediated ceRNA networks in cancer are continuously emerging (43, 44). However, only for a very limited number, such as *Gas5* (45), *linc-RoR* (46, 47), *NORAD* (48), and *linc-NeD125* (49), this function has been characterized: their aberrant enrichment or local increased concentration in pathological conditions can culminate in tumorigenesis. Finally, some lncRNAs may contain short open reading frames producing small, functional peptides (50).

## The role of lncRNAs in CNS

The CNS of mammals is a very sophisticated system in which neuronal and glial cells structurally and functionally interact to guarantee the proper brain activity. Numerous evidence correlates the evolutionary increase in human brain complexity with the expanding number of lncRNAs (51, 52). Accordingly, 40% of human annotated lncRNAs are expressed in the brain, where they display neuro-anatomical and/or cell-type specific expression, and about 30% of lncRNAs appears to be primate-specific (31, 53). Notably, compared to lncRNAs from other tissues, the brain-specific lncRNAs are: (i) the most evolutionarily conserved species, (ii) predicted to retain conserved secondary structures, and (iii) preferentially adjacent to protein-coding genes involved in neuronal differentiation and function (54). Overall, these findings indicate that brain-specific lncRNAs likely possess conserved functions and are crucially implicated in higher-order cognitive abilities as well as in establishing neural cell-type diversity and function. This hypothesis is sustained by their spatiotemporal expression, which is exquisitely regulated during NS development (55) and in response to neuronal activity (56). So far, a growing body of literature shows that lncRNAs influence every step of neurodevelopment, from early stages of differentiation to synaptogenesis (57–59). *In vitro* studies revealed some lncRNAs, such as *RMST* (60), *TUNA* (61), *DALI* (62), and *PAUPAR* (63), that control complex gene expression programs underlying the neurogenic commitment of pluripotent embryonic stem cells. This is mainly achieved through their action of “guide” RNAs that convey transcriptional and/or epigenetic factors on the promoters of neuronal genes. *In vivo* analyses identified other species such as *GOMAFU* (56, 64, 65), *EVF2* (66), *PNKY* (67), and *linc-BRN1B* (68) that, through the recruitment of epigenetic, transcriptional, or splicing factors, govern the balance between self-renewal and neuronal differentiation. LncRNAs also contribute to synaptogenesis and neuronal plasticity, which underlies learning, memory, and cognition, by regulating crucial proteins that control neurite elaboration (69), translation in synapses (70, 71), and ion channel subunits (72).

## lncRNAs in Neuro-oncological Disorders

Based on their crucial role in NS development and function, lncRNA qualitative and/or quantitative alterations may profoundly impact on different neurological pathologies, including neurodevelopmental, neurodegenerative, neuro-immunological, and neuro-oncological disorders (73, 74). In the latter settings, lncRNAs have drawn extensive attention as molecules that may drive tumorigenesis. In addition, they can serve as predictors of cancer sub-types as well as potential therapeutic targets.

It is widely understood that mutations, epigenetic alterations or somatic copy number aberrations in the noncoding portion of the genome underlie cancer pathology (75). Accordingly, recent studies indicated that lncRNAs are highly deregulated in cancer, where they participate as tumor-suppressors or oncogenes in tumor initiation and progression. Notably, most lncRNAs displaying aberrant expression are cancer-type unique (76). However, despite the identification of a large number of lncRNAs in neurological cancers, only for a few of them mechanisms of action have been experimentally clarified.

Extensive studies have been carried out in gliomas, the most prevalent types of primary intracranial carcinoma (77). Several lncRNAs associated with glioma stemness (78–80), proliferation, and migration (81–84) have been identified, and most of them function as miRNA decoys (81).

Neuroblastoma (NB) is a pediatric tumor of the sympathetic NS, accounting for more than 7% of childhood malignancies (85). The molecular link between deregulated lncRNA expression and NB tumorigenic features is emerging (86), and several deregulated lncRNAs during NB pathogenesis have been uncovered (87–92).

Our knowledge of lncRNA function in MB physiopathology is still fragmentary. Genome-wide association studies may help to understand how genetic polymorphisms in lncRNA loci contribute to MB predisposition (93). Furthermore, in spite of the numerous high-throughput expression studies carried out so far, lncRNAs have been largely disregarded. However, re-annotation of array-based data and integration of cancer phenotype associations allowed prioritizing disease-related lncRNAs in tumors, including MB (94), demonstrating the potential of data re-analyses. In another study, a *de-novo* genome-wide inspection of MB subgroup-specific chromosomal alterations identified the first G3 MB gene fusions (6). They involve the 5′-end of *PVT1*, a lncRNA hosting the putative MB oncogene miR-1024 (95, 96). In the *PVT1-MYC* fusion, the induction of miR-1024 and the associated malignant phenotype may be explained through an oncogenic positive feedback-loop, established by MYC on its response elements on PVT1 promoter (6).

Other studies focused on the role played in MB by previously identified noncoding oncogenes. Among them, *UCA1* (97) and *CRNDE* (98, 99) are upregulated in MB samples. *UCA1* knockdown in MB cells results in the arrest of cell cycle progression, suppression of cell migration, and proliferation (100). Similarly, *in vitro* downregulation of *CRNDE* blocked cell cycle, inhibited proliferation and aggregation, while increasing apoptosis. Tumor growth was also reduced in MB mouse models silenced for CRNDE (101). Inversely, the lncRNA *HOTAIR* (102) is downregulated in MB samples, whereas its target genes *HOXD8* and *HOXD10* are upregulated (103). The misbalance of these crucial developmental genes may partially account for the embryonic origin and the pediatric onset of MB. However, their mechanisms of action are presently unknown.

Mechanistic insights into the role of lncRNAs in MB biology have been carried out only for a very few species, as discussed below.

### Mechanisms of Action of lncRNAs in Mb

The colon cancer upregulated transcript *CCAT1* (104) is a prototype of oncogenic lncRNA, associated with several carcinomas, where it promotes cell proliferation, invasion, migration, and chemoresistence (105–107). In MB, its expression is upregulated in 20 unstratified tumor samples and also in at least four MB cell lines (108). *CCAT1* knockdown in MB cells causes the decrease of cell proliferation rate, (depending on *CCNA* and *CDK2* gene repression), cell migration, and invasion. Its *in vivo* depletion reduces the volume of subcutaneous tumors of xenotransplanted mice (108). *CCAT1* has been proposed to play its oncogenic role by altering the phosphorylated, active status of components of the tumorigenic MAPK pathway. In combination with previous reports indicating *CCAT1* as a miRNA sponge (109–111), this study suggests that *CCAT1* may control tumorigenesis through multiple activities.

Another lncRNA implicated in MB is *ANRIL* (112), which plays a pivotal role in multiple cancers as an epigenetic regulator of its neighbor tumor-suppressors *CDKN2A/B* (113, 114). *ANRIL* expression is upregulated in MB cells, where its knockdown lowers cell viability and migration while increasing apoptosis, by deranging the expression of several apoptotic factors (115). *ANRIL* has been shown to act as a decoy for miR-323, a miRNA identified in neurons (116) and characterized as a glioma tumor-suppressor (117, 118). Consistently, miR-323 silencing counteracted the abovementioned *ANRIL*-dependent cell phenotypes. This regulative axis impinges on *BRI3*, a miR-323 target gene (119) encoding for a brain-expressed transmembrane factor (120). *BRI3* activates MAPK, AKT and WNT signaling cascades, already associated with MB progression (121–123), through a double mechanism: *BRI3* upregulation enhances the phosphorylation of p38, MAPK, ERK, and AKT kinases and stimulates the accumulation of Wnt3a, Wnt5a, and β-catenin. The dysregulation of such pathways may partially explain the apoptotic phenotypes observed upon imbalance of *ANRIL*/miR-323/*BRI3* module (115).

More recently, the lncRNA *LOXL1-AS1*, the antisense transcript to the LOXL1 genomic locus, whose variants are strongly associated with the exfoliation syndrome (124), was found to be overexpressed in MB tissues. *In vitro* and *in vivo* experiments revealed that it controls cell viability, proliferation, cell cycle, and metastasis by activating the PI3K-AKT pathway (125).

**Figure 2:**
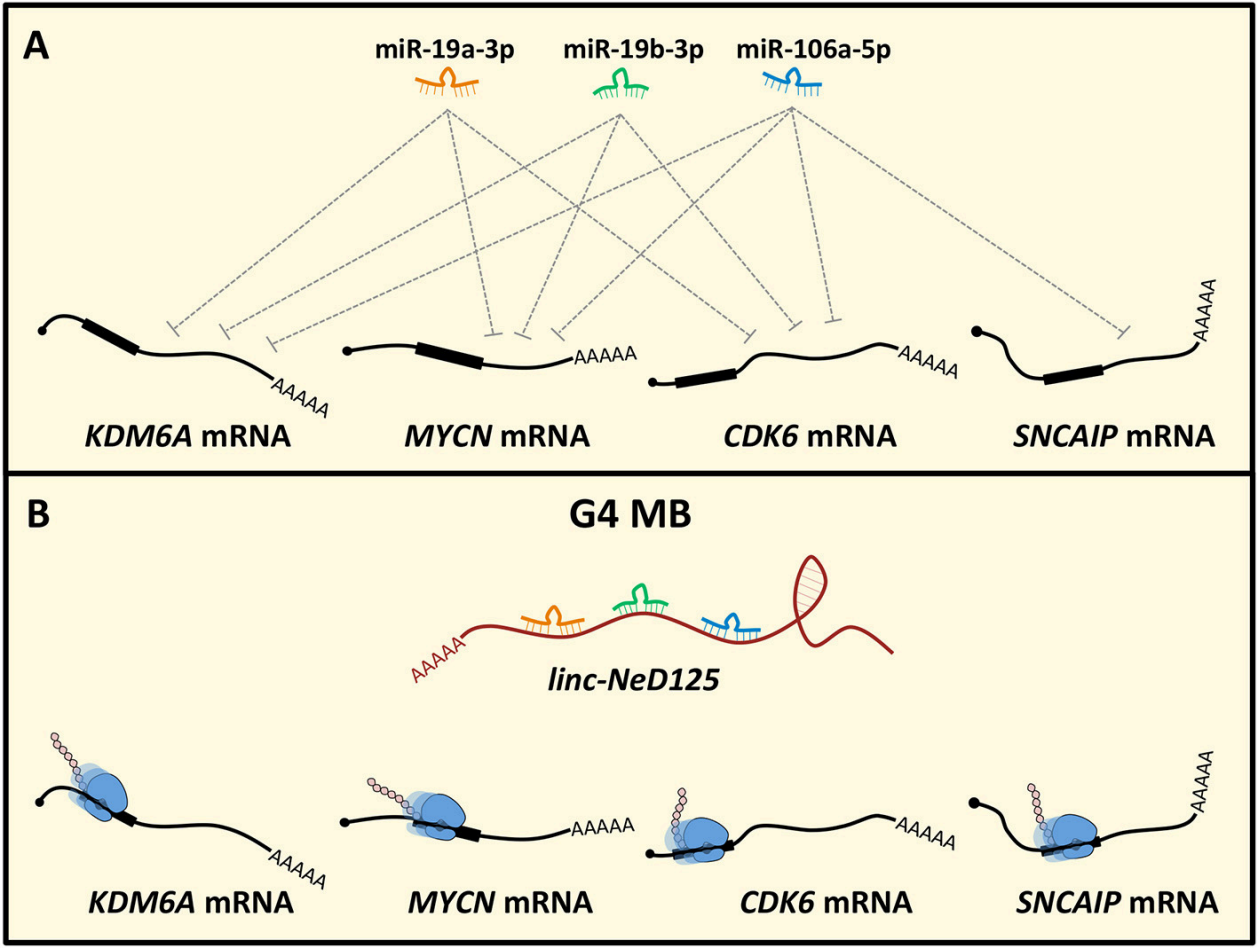
*Linc-NeD125*-based network in MB. **(A)** MiR-19a-3p, miR-19b-3p, and miR-106a-5p pleiotropically control *KDM6A, MYCN, CDK6, SNCAIP* gene expression. **(B)** In G4 MB, *linc-NeD125* is upregulated and, functioning as a decoy for the three miRNAs, causes translational derepression of the G4 MB driver genes *KDM6A, MYCN, CDK6, SNCAIP*.

Recently, the ceRNA mechanism has emerged as a crucial pathogenic pathway in MB. *Linc-NeD125* was the first ceRNA identified in MB and, generally, in tumors of the CNS (49). It was identified in NB cells as the precursor of miR-125-b1 (126), a neuronal-enriched miRNA (127) involved in neural cell differentiation (128), function (129) and NB and MB cell proliferation, and apoptosis (130, 131). Notably, *linc-Ned125* is significantly and specifically upregulated in primary G4 MBs, compared to the other subgroups. In this context, it functions as a miRNA decoy. *Linc-NeD125* interacts with miR-19a-3p, miR-19b-3p, and miR-106a-5p that pleiotropically control the expression of four G4 MB driver genes, namely *KDM6A, MYCN, CDK6*, and *SNCAIP* (7) (Figure 2). Through this mechanism, *linc-NeD125* causes the driver gene translational de-repression, contributing to G4 MB tumorigenesis and/or to the maintenance of cancer cell identity. This study highlighted *linc-NeD125* as a novel potential G4 driver gene, as well as a specific biomarker and a potential therapeutic target. Accordingly, its knockdown in G4-derived cells caused a significant reduction of cell proliferation, migration, and invasion (49).

The second example of ceRNA in MB is the lncRNA *Nkx2-2as*, that behaves as a tumor-suppressor in SHH MB subgroup. It is highly down-regulated in MB cells derived from a SHH mouse model and it suppresses the malignant phenotype of MB cells, functioning as a sponge for miR-103/107 and miR-548 m. This activity causes the depression of the tumor-suppressor genes *BTG2/Tis21/PC3* and *LATS1/2*, promoting tumor growth *in vitro* and *in vivo* (132).

## Future Directions

The main challenges in fighting cancer are the identification of specific biomarkers, for timely diagnosis and prognosis, and novel tumor-driver genes, which can be therapeutically targeted for suppressing tumor growth. The former function would help the choice of pre-operative treatments and facilitate the tumor follow-up examinations. Unfortunately, very few biomarkers are known for pediatric tumors (133) and in MB <20 protein-coding genes have been characterized as promising candidates. However, most of these biomarkers were identified from single studies and from heterogeneous tumor types, lacking tumor-specificity (133). The recent categorization of MB into at least four subtypes, with distinct features, led the scientists to consider them as distinct pathologies with likely different responses to therapy. This new perspective triggered the search for novel MB-subgroup specific biomarkers and therapeutic targets. For both issues lncRNAs are very challenging (75). Since many of them are uniquely expressed in specific cancer types, they may function as powerful cancer biomarkers (134). In addition, for their ability to fold into complex tridimensional structures that increase their stability, they can be easily detected into body fluids as urine, blood, and cerebrospinal fluids, making the tumor diagnosis less invasive (75). Notably, lncRNAs are also considered new relevant targets for cancer therapy as highly tissue-specific drivers of cancer phenotypes. Finally, in this search for lncRNAs as novel molecules that distinguish clinically relevant cancer subtypes and predict tumor behavior, the circular RNAs are proving to be effective cancer biomarkers for their abundance, stability, and specificity (135).

## Author Contributions

PL and EC reviewed the literature and wrote the manuscript. JR prepared the figures and contributed to the text discussion.

### Conflict of Interest Statement

The authors declare that the research was conducted in the absence of any commercial or financial relationships that could be construed as a potential conflict of interest. The handling editor declared a past co-authorship with the authors PL and EC.
